# *In Vitro* and *In Vivo* Evaluations of PLGA Microspheres Containing Nalmefene

**DOI:** 10.1371/journal.pone.0125953

**Published:** 2015-05-04

**Authors:** Xiangyang Xie, Wen Lin, Chuanfeng Xing, Yanfang Yang, Qiang Chi, Hui Zhang, Ying Li, Zhiping Li, Yang Yang, Zhenbo Yang, Mingyuang Li

**Affiliations:** 1 Department of Pharmacy, Wuhan General Hospital of Guangzhou Military Command, Wuhan, PR China; 2 Department of Clinical Laboratory, Huangshi Love & Health Hospital of Hubei province, Huangshi, PR China; 3 Dalian Merro Pharmaceutical Factory, Dalian, PR China; 4 Beijing Key Laboratory of Drug Delivery Technology and Novel Formulation, Institute of Materia Medica, Chinese Academy of Medical Sciences & Peking Union Medical college, Beijing, PR China; 5 Department of Pharmacy, The 215th Clinic of 406th Hospital of the Chinese People's Liberation Army, Dalian, PR China; 6 Department of Pharmaceutics, Beijing Institute of Pharmacology and Toxicology, Beijing, PR China; University of Akron, UNITED STATES

## Abstract

Poor patient compliance, untoward reactions and unstable blood drug levels after the bolus administration are impeding the pharmacotherapy for insobriety. A long-acting preparation may address these limitations. The aim of this paper was to further investigate the *in vitro* characteristics and *in vivo* performances of nalmefene microspheres. Nalmefene was blended with poly (lactide-co-glycolide) (PLGA) to prepare the target microspheres by an O/O emulsification solvent evaporation method. The prepared microspheres exhibited a controlled release profile of nalmefene *in vitro* over 4 weeks, which was well fitted with a first-order model. *In vitro* degradation study showed that the drug release *in vitro* was dominated by both drug diffusion and polymer degradation mechanisms. Pharmacokinetics study indicated that the prepared microspheres could provide a relatively constant of nalmefene plasma concentration for at least one month in rats. The *in vivo* pharmacokinetics profile was well correlated with the *in vitro* drug release. Pharmacodynamics studies revealed that the drug loaded microspheres could produce a long-acting antagonism efficacy on rats. These results demonstrated the promising application of injectable PLGA microspheres containing nalmefene for the long-term treatment of alcohol dependence.

## Introduction

Alcohol dependence is a major public health issue throughout the world. Despite its high prevalence, the management of alcohol dependence still remains inadequate. It was reported that there was only 8% of patients with alcohol dependence or alcohol abuse received formal treatments in Europe, and the corresponding figure was around 25% in the United States of America (USA) [1,2]. After a period of alcoholism with or without recommended therapy, quite a few patients will go back to alcohol drinking within 3–5 months [3,4]. In short, alcohol dependence is a chronic, relapsing disorder [4].

Currently, the therapy for alcohol dependence can be achieved mainly by combining psychosocial management with pharmacological interventions [5,6]. There are a few drugs available for pharmacotherapy of alcohol dependence, for example, the anti-craving substances naltrexone and acamprosate, and the aversion therapeutic agent disulfiram [7]. Among these medicines, nalmefene is considered to be a milestone in the advancement of alcohol dependence treatment. It is the first drug that approved in the European to reduce alcohol use in alcohol-dependent patients in 2013 [8]. Nalmefene possesses the antagonistic properties at the μ- and δ-receptor and partial agonistic properties at the k-receptor [9]. Compared with naltrexone, nalmefene has the advantages such as longer half-life (t_1/2_ ~10 h), greater oral bioavailability and lower dose-dependent liver toxicity [10,11].

Although the oral administration has been demonstrated to be effective in the management of alcohol dependence, successful pharmacotherapy is still limited by the poor patient adherence to the daily dosing schedule [12,13], fluctuations of blood drug levels [14], and adverse effects at the required doses [15,16]. These factors may result in the interrupted therapy or premature discontinuation [17] and thus lead to the failure of the pharmacological treatments. As for nalmefene, the present available dosage form in the market is tablet and injection, these bolus administrations need frequent dosing and may produce a high peak blood concentration, thus result in the poor medication compliance of alcohol-dependent patients. To address these limitations, a long-term drug delivery system, which can deliver a lower constant dose of nalmefene for a long time, offers an option to overcome such constraints. Accordingly, Costantini et al. prepared an implantable ethylene vinyl acetate (EVA) rod containing nalmefene for sustained release [14]. However, this EVA rod is non-erodible *in vivo* and needs to be withdrawn from the body when the drug is run out. Therefore, a biodegradable injectable delivery system may solve the non-erodible problem existing in EVA rods.

Injectable and biodegradable microspheres were widely investigated and studied in the past decades. Such microspheres can extend the drug acting duration remarkably, decrease the dosing frequency and thus better the compliance of patient. The drug dose and certain adverse effects could be diminished for the stable blood drug levels the microspheres will bring [18]. Moreover, there is no need to implant in and remove out these microspheres through surgical operation during the drug delivery period. Poly (lactic-co-glycolic acid) (PLGA), the US Food and Drug Administration (FDA) approved biodegradable polymer for *in vivo* use, is now employed in several parenteral microspheres in the market (e.g. Zoladex, Trelstar, Vivitrol, Lupron Depot and Risperdal Consta). As PLGA has a good biocompatibility and sustained drug delivery profile, it can be used in the development of an injectable microsphere system, which is able to continuously delivery nalmefene *in vivo* for a long term. Although a similar idea was reported by Wu et al. (for the treatment of opioid dependence) [19], the *in vitro* release mechanism and *in vivo* performance of nalmefene microspheres remain superficial and worth a detail study.

In this paper, an extended-release PLGA microsphere for nalmefene was prepared and appraised. An O/O solvent evaporation technique was utilized to prepare the microspheres, and then the physicochemical properties and release profile *in vitro* of the microspheres was evaluated. Liquid chromatography-mass spectrometer/mass spectrometer (LC-MS/MS) was used to determinate the nalmefene concentration of the plasma samples. After obtained the pharmacokinetics data, an *in vitro-in vivo* correlation (IVIVC) of the prepared microspheres was estimated. In the end, the preliminary pharmacodynamics and immunotoxicity character of microspheres were investigated.

## Materials and Animals

### Ethics Statement

This study did not involve non-human primates. All animal related experiments were carried out under the guidelines of Care and Use of Laboratory Animals released by the National Institute of Animal Health. This study is approved by the Animal Care and Use Ethics Committee of the 215th Hospital (ETHICS CODE Permit NO.SCXK (Liao) 2014–0047).

### Materials

PLGA (molecular weight 20000 Da, lactide/glycolide ratio, 75/25) were purchased from the Institute of Chemistry (Wuhan University, China) in their hydrophilic forms (carboxylic acid end group). Nalmefene and Nalmefene Hydrochloride (> 99% purity) were supplied by Xian-Lijun Ppharmaceutical Company (Shanxi, China). Nalbuphine (99.6% purity), the internal standard, was purchased from Sigma (St. Louis, MO, USA). Morphine injections were obtained from Qinghai Pharmaceutical Plant (Qinghai, China). Liquid paraffin, dichloromethane (DCM) and acetonitrile (AN) were purchased from Beijing Chemical Reagents Company (Beijing, China). Tween-80 was supplied by Fisher Scientific (Hongkong, China) Ltd. All other materials or solvents were of reagent or analytical grade.

## Experiment and Methods

### Preparation of microspheres

An O/O emulsification solvent evaporation approach based on the description of Wu et al. [19] was applied to prepare the microspheres. Briefly, 180 mg of PLGA and 15 mg of nalmefene were added and dissolved in 1 mL of DCM-AN solvent (1: 1, v/v). Then, the mixture was poured quickly into 20 mL of liquid paraffin containing Span-80 (1.5%, w/v) as emulsifier, and emulsified through a propeller stirrer (SXJQ-1, Zhengzhou, China) at 650 rpm for 10 min at the temperature of 25°C. To evaporate the organic solvent in the O/O emulsion, the stirring speed was changed to 450 rpm and kept for 10 h. By filtering through a filter-paper, the solidification microspheres were gathered, rinsed 3 times with 15 mL hexane and then washed with 20 mL deionized water. Finally, the collected microspheres were dried at a vacuum chamber under room temperature.

### Drug loading and entrapment efficiency

Accurately weighed 25 mg nalmefene microspheres were dissolved in 2 mL of an AN-water solution (9: 1, v/v) and followed by a 10 fold dilution with 0.001 mol·L^-1^ HCl. The resulting solution vortexed for 2 min and then kept undisturbed for 5 min. After centrifuged with a speed of 10,000 rpm for 10 min, the supernatant was collected and its nalmefene concentration was analyzed [20] by a high performance liquid chromatography (HPLC) system (Waters 2487, Waters, USA). The HPLC consisted of a pump and a ultra-violet and visible light (UV-Vis) detector with the detective wave of 284 nm. A reversed phase C_18_ column (5 μm, 4.6 mm × 250 mm, Agela technologies, China) was used and its analysis temperature was set as 25°C. KH_2_PO_4_ aqueous solution (pH 4.0; 0.02 M), methanol and triethylamine (60: 40: 0.2, v/v/v) were mixed and used as the mobile phase at the flow rate of 1 mL/min. The injection volume was 20 μL.

The drug loading (DL, %) and encapsulation efficiency (EE, %) were calculated by the following equations [21]:

DL = (drug found in microspheres/microspheres weight)×100%

EE = ( drug found in microspheres/drug added) ×100%

### Particle sizing

The average particle size and size distribution of the prepared microspheres were determined by a light-scattering particle size analyzer (Ls800, OMEC, China). Briefly, microspheres were suspended in 0.5% (w/v) sodium carboxymethyl cellulose (CMC-Na) solution [22] and then analyzed.

### Morphological characterization

To investigate the morphological characterization of the prepared microspheres, the prepared microspheres were mounted onto a double-sided adhesive tape attaching to an aluminum stub, then coated with gold and examined by a scanning electron microscopy (SEM, Hitachi S-4800, Japan) at 5 keV acceleration voltage and 15 mm working distance.

### *In vitro* release assay

Studies on *in vitro* release of nalmefene from the microspheres were carried out in 30-mL cylindrical tubes containing 25 mL of phosphate buffer solution (PBS, 0.1 M, pH 7.4), 0.02% sodium azide (w/v) and 0.5% sodium dodecyl sulphate (SDS, w/v). In each tube, 10 mg of prepared microspheres were added. The tubes were incubated in a waterbath at 37 ± 0.5°C and vibrated horizontally at a speed of 72 rpm [23]. The following operations were taken as we previously described [24]. The nalmefene concentrations were assayed by the HPLC.

### *In vitro* mass loss

This study was conducted in the same processes as mentioned above. At each sampling time point (correspond to the time intervals used for the *in vitro* release study), the corresponding tubes were withdraw and the supernatant and the sediment were collected separately by centrifugation at 5,000 rpm for 5 min. The supernatant aqueous phase was removed and collected for gel permeation chromatographic (GPC) analysis, while the sediment microspheres were rinsed with distilled water, dried for 48 h at vacuum under room temperature and weighed [25]. The mass remaining of the microspheres was calculated by the following equation:

$$Percent\;mass\;remaining\;=\;\left(M_{2}/M_{1}\right)\times 100\%$$

where M_1_ represents the initial weight of microspheres and M_2_ denotes the weight of degraded samples at predetermined time.

### Molecular weight determination of PLGA

Molecular weights of the degraded fragments for the prepared microspheres were characterized by a gel permeation chromatography (GPC, 1515 Isocratic HPLC Pump, Waters, USA) with a refractive index detector (2414 Refractic Index Detector, Waters, USA). Waters analytical columns Styragel HT2 (7.8 × 300 mm) and Styragel HT3 (7.8 × 300 mm) were used in the assay. Tetrahydro-furan (THF) was used as the mobile phase at a flow rate of 0.7 mL/min under 35°C. Samples from the supernatant of the degradation studies were filtered through a 0.45 μm filters and then injected in the GPC. The residual copolymers in the microspheres degradation progressed were dissolved in THF at the concentration of 0.05–0.2% (w/v) and filtered through a 0.45 μm membrane filter, determinate by the same separation conditions [26]. The injection volume was 20 μL. In the analysis, polystyrene standards (Mw from 480 to 950,000) were utilized to calculate the average molecular weights.

### Biocompatibility study

Methyl thiazolyl tetrazolium (MTT) assay described by Wu et al. with modification was applied in this study to determinate the biocompatibility of the microspheres [23]. In brief, blank microspheres suspended in Dulbecco’s modified Eagle’s medium (DMEM) at the concentration of 0.1 g/mL were incubated under 37°C for 24 h, then the suspensions were filtered through a 0.22 μm membrane to obtain the extracts. L929 mouse connective tissue fibroblast cell (supplied by the Cell Resource Centre of China, IBMS, CAMS/PUMC) was maintained (37°C, 5% CO_2_) in DMEM which contained 10% fetal bovine serum, 100 IU/mL penicillin, 2 mM glutamine and 100 μg/mL streptomycin. The following operational approaches of this experiment please see in the reference [24]. A Microplate Reader (Model 680, BIO-RAD, USA) was used to read the absorbance of each well at the wave length of 570 nm. The relative cell viability (%) was calculated by the following equation:

$$Relative\;cell\;viability\;\left(\%\right)=\;{\left[Absorbance\right]}_{test}/{\left[Absorbance\right]}_{control}\times 100\%\text{.}$$

### Animals and Treatments

58 male Sprague-Dawley rats (220 ± 20 g, 6 weeks), 50 female Kungming mice (20 ± 2 g, 5 weeks) and 24 male EWG/B guinea pigs (400 ± 50 g, 8 weeks) were supplied by Laboratory Animals Center of the 215th Hospital (China). All animals were treated under the guidelines of Care and Use of Laboratory Animals released by the National Institute of Animal Health. All animals were free to food and water and were housed in polypropylene cages under a 12 hour light and 12 hour dark photoperiod. The temperature of the vivarium was kept at 22 ± 3°C and the relative humidity level was maintained at 50 ± 5%. The condition (as activity status, eating situation and weight) of the animals was monitored twice per day during the studies. There were no unintended deaths of animals during both pharmacokinetics and pharmacodynamics studies. When the corresponding experiment was completed, the animals were anesthetized by 2% pentobarbital solution (40 mg/kg) through peritoneal injection and sacrificed by cervical dislocation 10 min later.

### Pharmacokinetics study

The microspheres for *in vivo* studies were sterilized by ^60^Co radiation at a dose of 10 kGy [27], which would induce less than 1% drug content lost, but had negligible influence on the drug release profile of microspheres.

12 male Sprague-Dawley rats (220 ± 20 g, 6 weeks) were randomly divided into two groups each of 6 rats. In the control group, nalmefene solution (diluted with sterile water) was subcutaneously injected at a dose of 3 mg/kg [14]. In the test group, the microsphere suspensions (90 mg nalmefene/kg) were subcutaneously injected into the back rat by a 21 gauge needle. The microspheres were dispersed in 2 mL water for injection (containing 0.05% Tween-80 and 0.5% CMC-Na) before the administration.

Blood samples about 0.8 mL were transported into tubes included heparin before the injection and at 0.03, 0.08, 0.25, 0.5, 0.75, 1, 2, 3, 5, 7, 12, 24, 30 h after the end of the drug administration for the control group, and 0.5, 1, 2, 3, 5, 7, 14, 21, 28, 35 d for the test group. Before the sampling, the rats were anesthetised (pentobarbital, 40 mg/kg) and then blood was withdrawn from retroorbital plexus at designed time points. Plasma samples were obtained by centrifuging (4°C) the blood samples at 2,000 rpm for 10 min, then collected the plasma and frozen them at -20°C until analysis.

Chromatographic analysis to determine the plasma nalmefene concentration was performed using an Agilent 1200 series LC system coupling with tandem mass spectrometry (LC-MS/MS, Agilent Technologies, Santa Clara, CA, USA), as described by Fang et al [28]. The endogenous proteins did not affect the chromatographic determination of drug concentration. The calibration range was from 0.5 to 700 ng/mL. The inter- and intra-batch precisions were smaller than 15%. The lower limit of quantification (LLOQ) of the method for nalmefene was 0.5 ng/mL. The recoveries of the method were larger than 80%.

The data of plasma nalmefene concentrations versus time were processed by the software of WinNonlin 5.2 (Trial vision, Pharsight Corp., USA) using the non-compartmental model. The slope of the linear regression fit in the logarithm scale plasma concentrations versus time data for the last three measurable points was used to calculate the elimination rate constant (k_e_), and apparent elimination half-life (t_1/2_) was calculated as 0.693/k_e_.

The correlation between the drug released (%) *in vitro* and absorbed *in vivo* (F_a_) was calculated. The F_a_ was calculated by the Wagner-Nelson method as follows [29]:

$$F_{a}=\;\left(C_{t}/k_{e}+AUC_{0-t}\right)/AUC_{0-\infty }$$

### Pharmacodynamics study

Hot plate test was used to assess the antinociceptive effects of nalmefene as used in the similar evaluation of naltrexone microspheres with minor modification [30]. In brief, female Kungming mice (20 ± 2 g, 5 weeks) were individually placed on a hot plate (GJ-8402, Baishi Medical Electronics, Zhejiang, China) maintained at 55 ± 0.2°C, and the latency (response time, RT) was recorded from the start to the endpoint of licking, jumping, or shaking hind paws. To avoid the possible thermal injury associated with longer exposure times, the maximum response latency (MRL) was set to 60 s.

50 mice with RT <30 s were divided into five groups with 10 each. At the beginning, mice were subcutaneously injected with different dose of microspheres suspended in 0.5% CMC-Na solution, placebo microspheres for control group, and naltrexone microspheres equivalent to 40, 80, 160, and 320 mg naltrexone/kg for group A-D, respectively. Mice were s.c. injected with morphine (20 mg·kg^-1^, ED_95_) on designed time point, and RT was obtained 30 min after the injection. Finally, the degree of antagonism of a narcotic antagonist (naltrexone) to morphine was calculated by the following equation:

$$Antagonism\;\left(\%\right)\;=\;\frac{RT\;of\;morphine\;administration\;-\;RT\;of\;morphine\;plus\;antagonist}{RT\;of\;morphine\;administration\;-\;RT\;of\;blank\;control}\times 100\%$$

### Immunotoxicity study

In this study, active systemic anaphylaxis test and passive cutaneous anaphylaxis test were used to evaluate the immunogenicity of prepared microspheres. The following two tests were carried out as Wang et al. described with minor modifications [31].

24 male EWG/B guinea pigs (400 ± 50 g, 8 weeks) were randomly divided into 4 groups of each with 6. Group A was the negative control group and each guinea pig was administered with physiologic saline; Group B was the positive control group and administered with bovine serum albumin (10 mg/kg); Group C was given drug loaded microspheres (250 mg/kg, microsphere weight) and Group D was administered drug loaded microspheres (1250 mg/kg, microsphere weight). Each animal was by intramuscular injection. These treatments were repeated at day 1, 3, 5, 7 and 9, respectively. Two weeks after the last administration, similar treatments were repeated on each group, except the animals in Group B were treated with penile vein administration. After the treatments, animal’s response to the challenge was observed over 60 min, and the times of onset and disappearance of allergicreaction related symptoms were recorded, which include restlessness, piloerection, shaking, sneezing, nose scratching, shortness of breath, coughing, gasping, urination, lacrimation, defecation, rales, dyspnea, purpura, unsteady gait, jumping, convulsions, horizontal turn, tidal breathing and death. Due the long acting characteristic of the nalmefene microspheres, changes of symptoms were recorded for each animal daily and lasted for 30 days.

32 male Sprague-Dawley rats (220 ± 20 g, 6 weeks) were randomly divided into the following 4 groups with 8 in each group. Group A was the negative control group (physiologic saline, 1 mL/kg); Group B was the positive control group (bovine serum albumin, 10 mg/kg); Group C was given drug loaded microspheres (120 mg/kg, microsphere weight) and Group D was given with drug loaded microspheres at 600 mg/kg (microsphere weight). Another 8 rats were divided into the same groups with 2 rats in each group. Intramuscular injections were repeated 5 times every other day for sensitization, after that, blood samples were collected and centrifuged at 2000 rpm for 10 min to obtain serums and frozen at -20°C until analysis. Both sides of the back for each rat were shaved (3×4 cm^2^), and anti-serum (diluted with saline at the ratio of 1: 2, 1: 8 or 1: 32) of corresponding group was injected intradermally at two points for each diluted anti-serum (0.1 mL). 24 h after the injections, rats were injected with the corresponding drugs. 30 min later, 4 rats in each group were injected with Evans blue dye (0.5%, 1 mL) intravenously. The rats were anesthetized by 2% pentobarbital solution (40 mg/kg) and sacrificed by cervical dislocation 30 min later. The skin at the injection site of the anti-serum was collected by a 1cm punch, cut into pieces and placed into 5 mL of saline solution of acetone. Optical density (OD) values at 590 nm were determined 24 h later. After 30 d, the left 4 rats in each group were handled as before.

### Statistical analysis

Each measurement was repeated in triplicate. All data are presented as mean ± standard deviation (SD) unless especially denoted. A p < 0.05 was considered significant as determined by one-way analyses of variance (ANOVA) or a standard Student’s t test. All data were processed by the software of SPSS 11.0 (SPSS Inc., Somers, New York).

## Results and Discussion

### Morphology, particle size and drug loading

**Fig 1:**
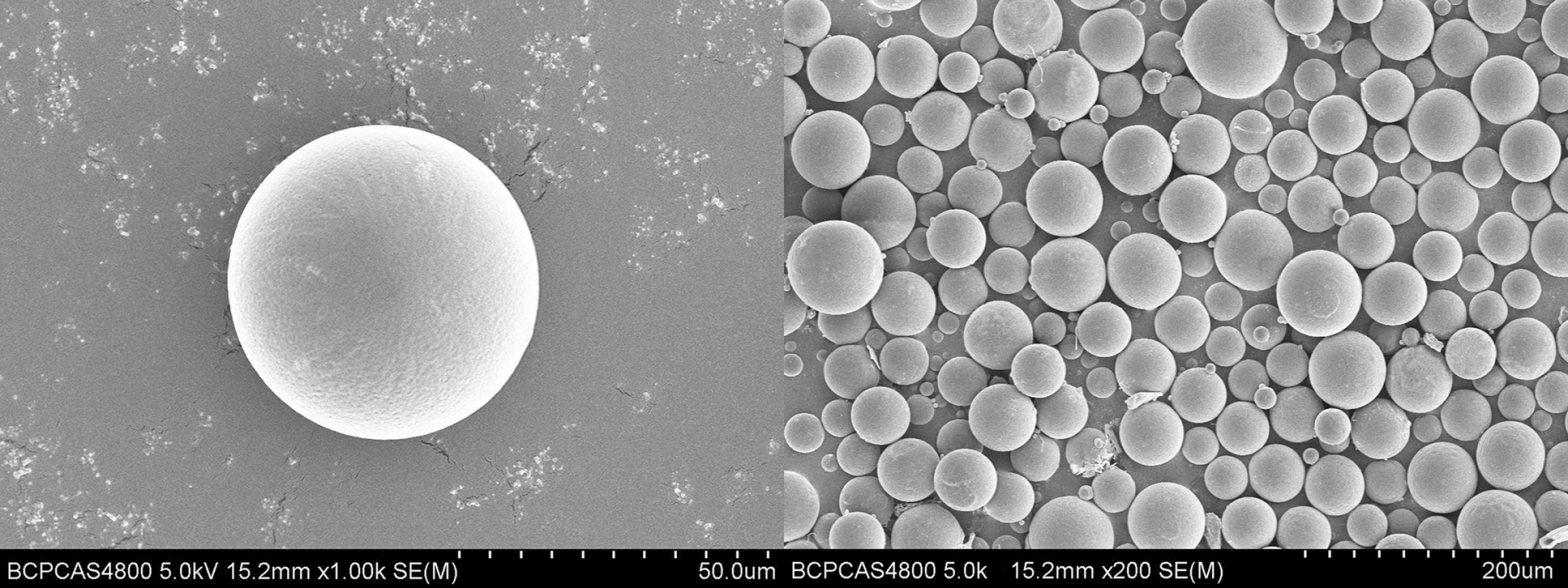
Scanning electron micrographs of nalmefene loaded PLGA microspheres.

It was shown in Fig 1 that the microspheres prepared in this study are regular and spherical with smooth surfaces.

**Fig 2:**
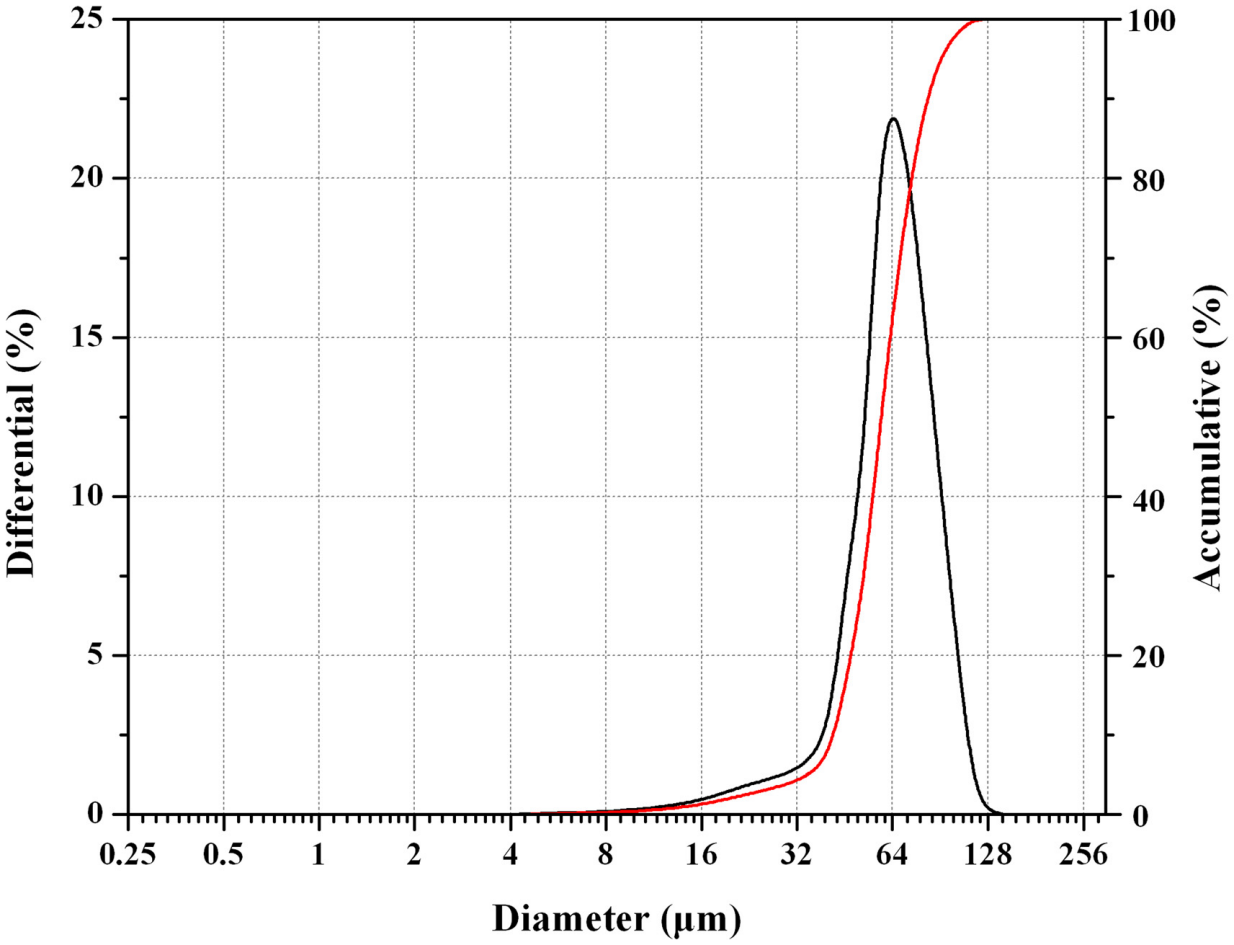
Particle size distribution of nalmefene loaded PLGA microspheres.

As displayed in Fig 2, the average microsphere size of prepared microparticles is around 60 μm, with an average value of 58.9 ± 2.1 μm. The span of size distribution was 1.02 ± 0.04 (n = 3), which implied that the particle diameter of the microspheres was uniform and homogenous. This result is consisted with the SEM observation mentioned above.

The drug content of the prepared microsphere was 6.84 ± 0.06% and the encapsulation efficiency was 78.74 ± 1.93 (n = 3).

According to a previous reference reported [19], the O/O solvent evaporation (SE) method was selected to prepare the microspheres here. There are many advantages for the method of SE, for example, it can be done under ambient temperature with constant stirring [32] and no special equipment was needed [33]. Thus, it is usually chosen and applied in the preparation of microspheres. In preliminary studies, nalmefene hydrochloride was used to prepare the microspheres by O/O, W/O/O, W/O/W, S/O/O and S/O/W solvent evaporation method, respectively. However, the drug encapsulation efficiencies of these methods were all less than 10%. To enhance the encapsulation efficiency, the relative more hydrophobic form-nalmefene was used with the current reported O/O method. This might because the hydrophobic nalmefene binds more tightly with lipophilic PLGA and thus generate a higher drug encapsulation [24].

### *In vitro* drug release

**Fig 3:**
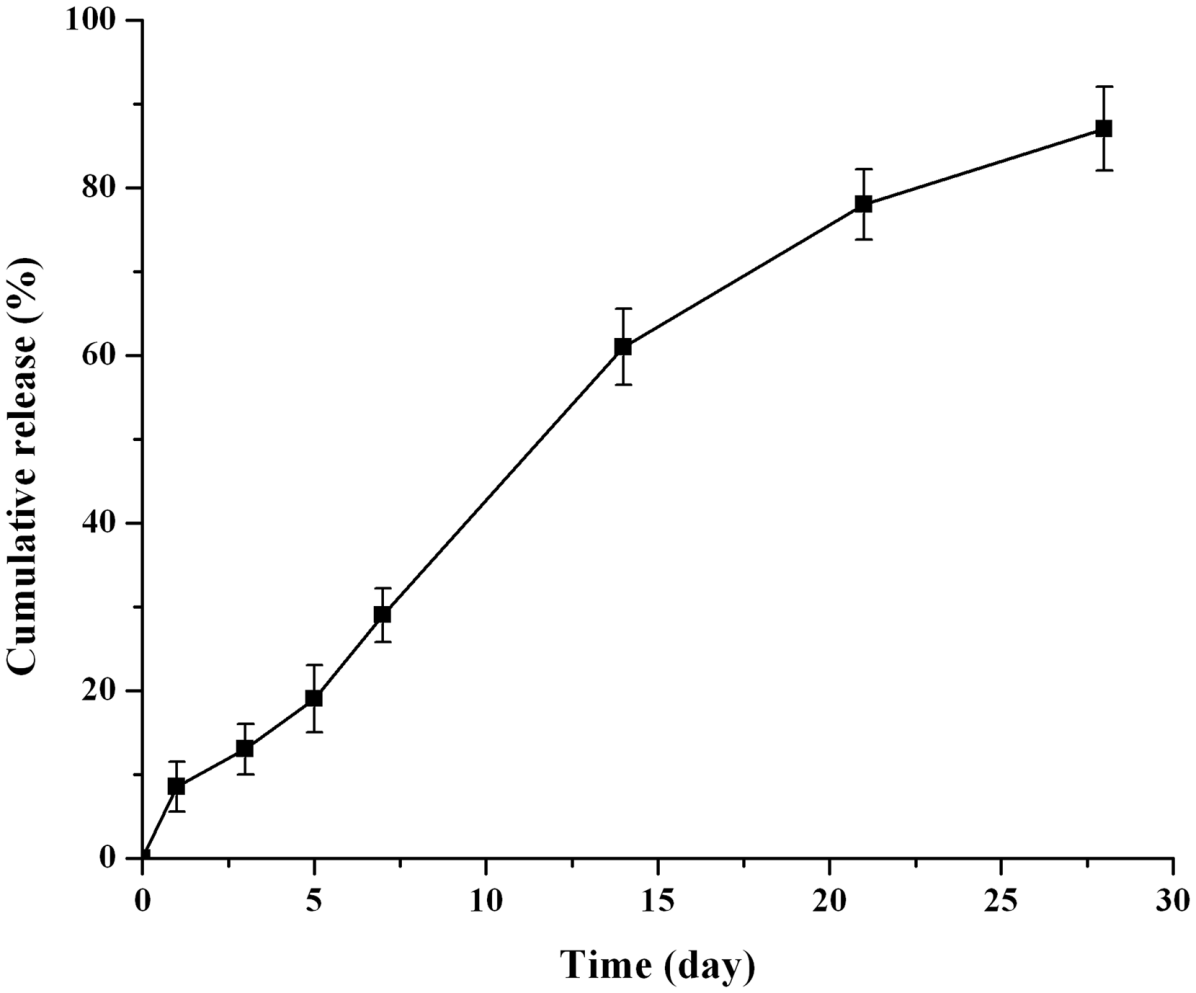
*In vitro* release profile of nalmefene loaded microspheres. In 0.1 M PBS (pH 7.4) at 37°C (n = 6).

The *in vitro* drug release cure (cumulative release versus time) of nalmefene loaded microspheres was demonstrated in Fig 3. Approximately 8% of the drug was release during the initial 24 h, then the drug release rate decreased within 2–7 day; the release rate increased again in the 7–14 day, and then it slowed down gradually to the end of the study. By the day 28, the cumulative drug release was more than 85%.

The above release data were fitted with different mathematical models and the following equations were obtained: zero-order (F_t_ = 3.249t + 4.849, R^2^ = 0.968), first-order (Ln(100 - Ft) = -0.074t + 4.680, R^2^ = 0.991) and Higuchi (Q = 18.18t^1/2^ - 11.31, R^2^ = 0.948). Obviously, the first-order equation was the best fitted model among the three equations because it had the highest correlation coefficient R^2^. This result may suggest that the diffusion may not be the major release mechanism for the *in vitro* nalmefene release [34]. The *in vitro* drug release mechanism will be further discussed in the following part.

### Mass loss

**Fig 4:**
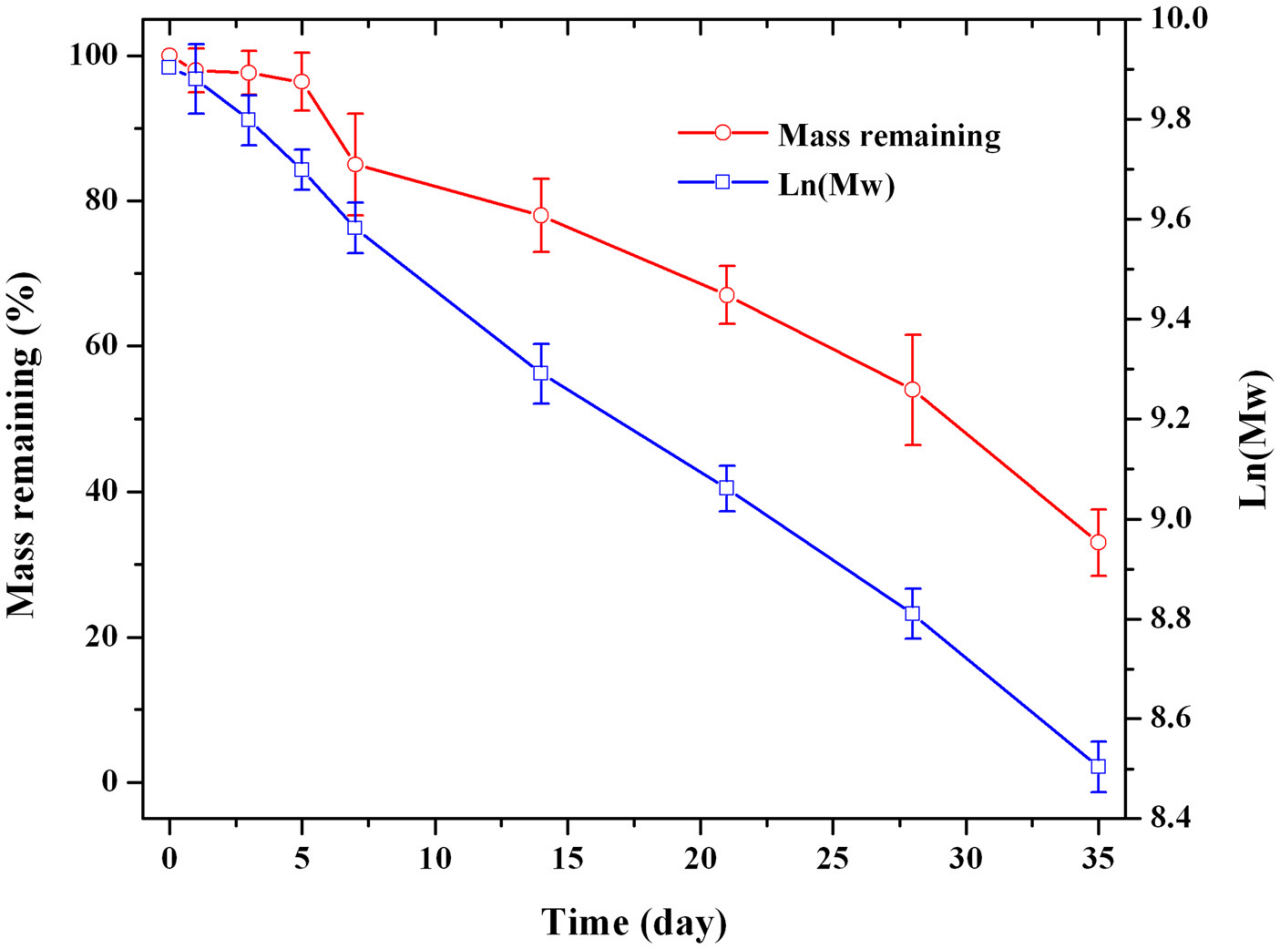
*In vitro* degradation study of nalmefene loaded PLGA microspheres. In 0.1M PBS (pH 7.4) at 37°C (n = 6). Mw represents molecular weight.

To elucidate the *in vitro* drug release mechanism of the prepared microspheres, *in vitro* polymer degradation experiments were carried out. The *in vitro* mass loss behavior and molecular weight declining profile of the PLGA microspheres were displayed in Fig 4.

During the first 5 days, the mass remaining kept almost constant. Then, the mass of microspheres initiated to decrease, losing about 67% weight at the day 35. While, initiated from the first day, the molecular weight (Mw) of PLGA decreased as the time passage. As Ln(Mw) and time correlated well in a line (Ln(Mw) = -0.036t + 9.870, R² = 0.985), the polymers demonstrated a pseudo-first-order degradation [35]. The above result implied that the PLGA degradation phenomenon was occurred during the polymer mass loss process.

At the first day of *in vitro* release test, an initial large bolus of drug was released due to the drugs on the surfaces of microspheres diffused out [21]. In the initial five days, water soluble oligomers with low molecular weights might have been generated [36], and the drug release mechanism was mainly governed by diffusion during this period. After that, the oligomers left the PLGA matrix and it initiated the mass loss process, thus it lead to a rapid drug release. In final stage, the nalmefene release slowed down, the drug release was dominated by both diffusion and polymers degradation [37]. Over all, the drug release mechanism of nalmefene-loaded PLGA (20 kDa) microspheres was combined with drug diffusion and polymer degradation. The dominant release mechanism would change during different release stage.

### Biocompatibility

**Fig 5:**
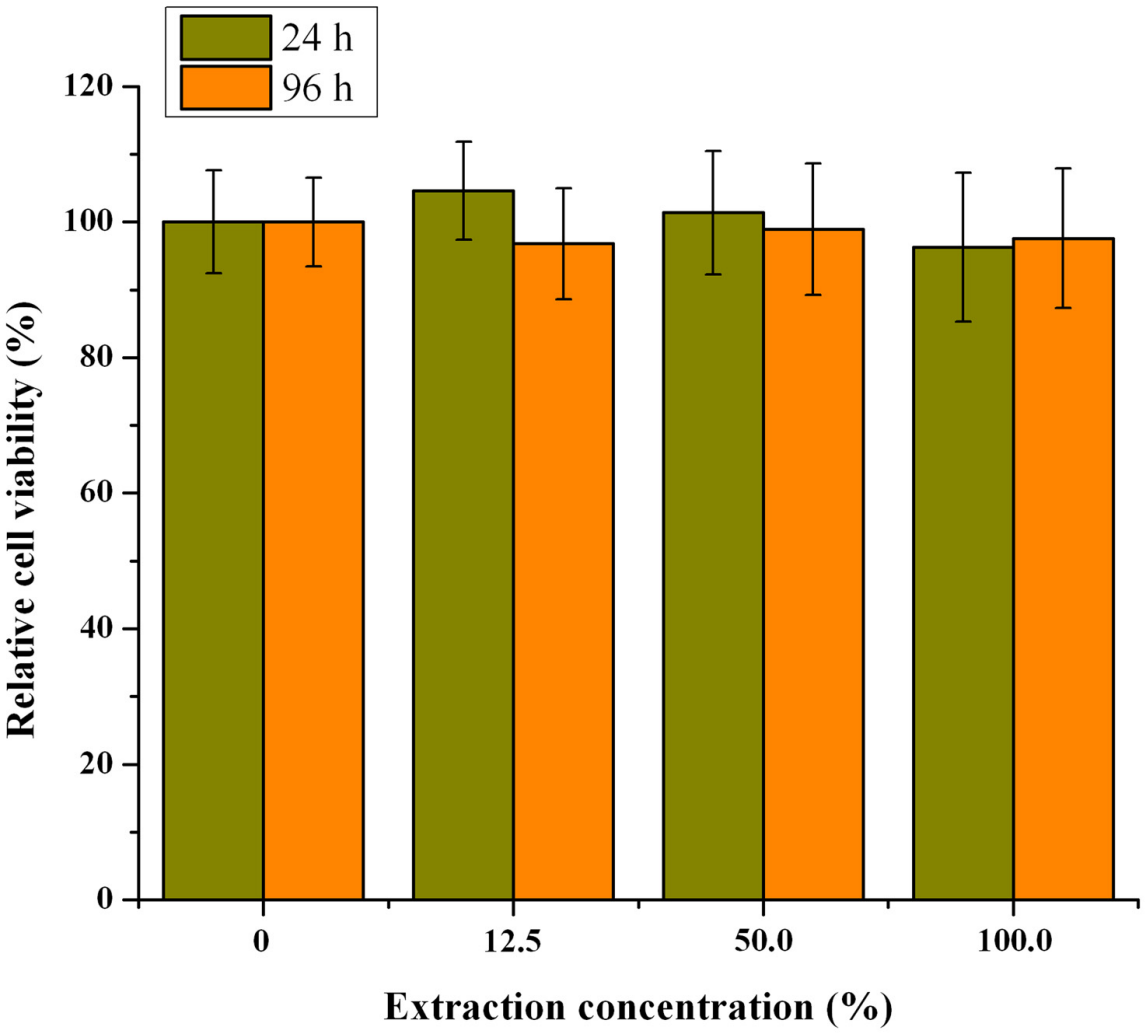
The MTT results for the biocompatibility of PLGA microspheres at different extraction concentrations (n = 6).

Biocompatibility is a key issue for materials used *in vivo* and needs special attention. Even though the excellent biocompatibility of PLGA polymer were widely reported by references [28], it is still essential to investigate the potential toxicity of the prepared microspheres. Here, the biocompatibility of the prepared microparticles was estimated through a MTT test. As showed in Fig 5, using the relative cell viability as the index, there were no significant difference between the control and test groups at the both testing time points of 24 and 96 h (p > 0.05). All the relative cell viabilities of microspheres groups were greater than 80%, so this result suggest that the prepared microspheres exhibited low or no cytotoxicity [27] and could be used for *in vitro* drug delivery.

### Pharmacokinetic

**Fig 6:**
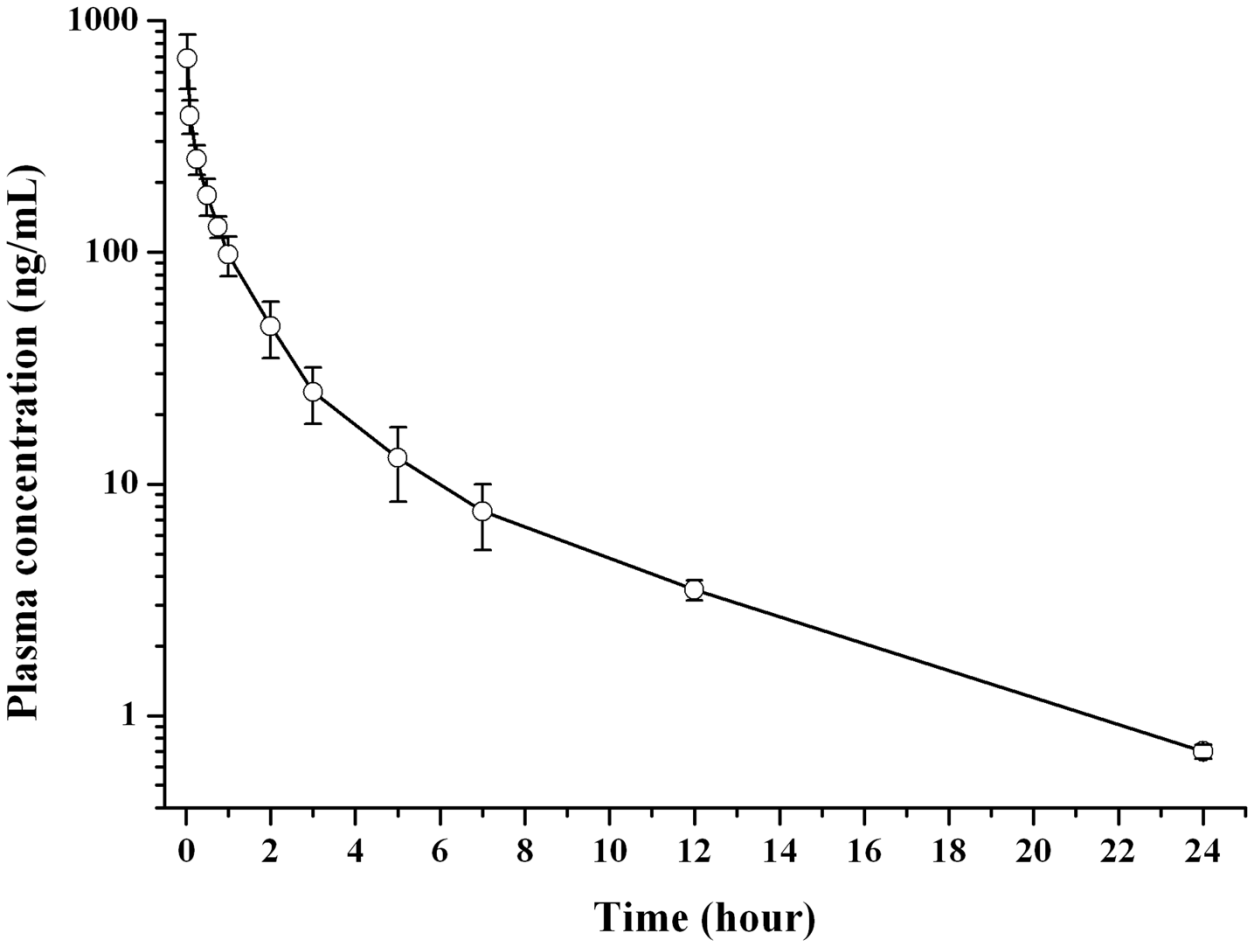
Mean plasma drug concentration-time curve of nalmefene in rats following the single subcutaneous injection of drug solution in rats (n = 6).

**Fig 7:**
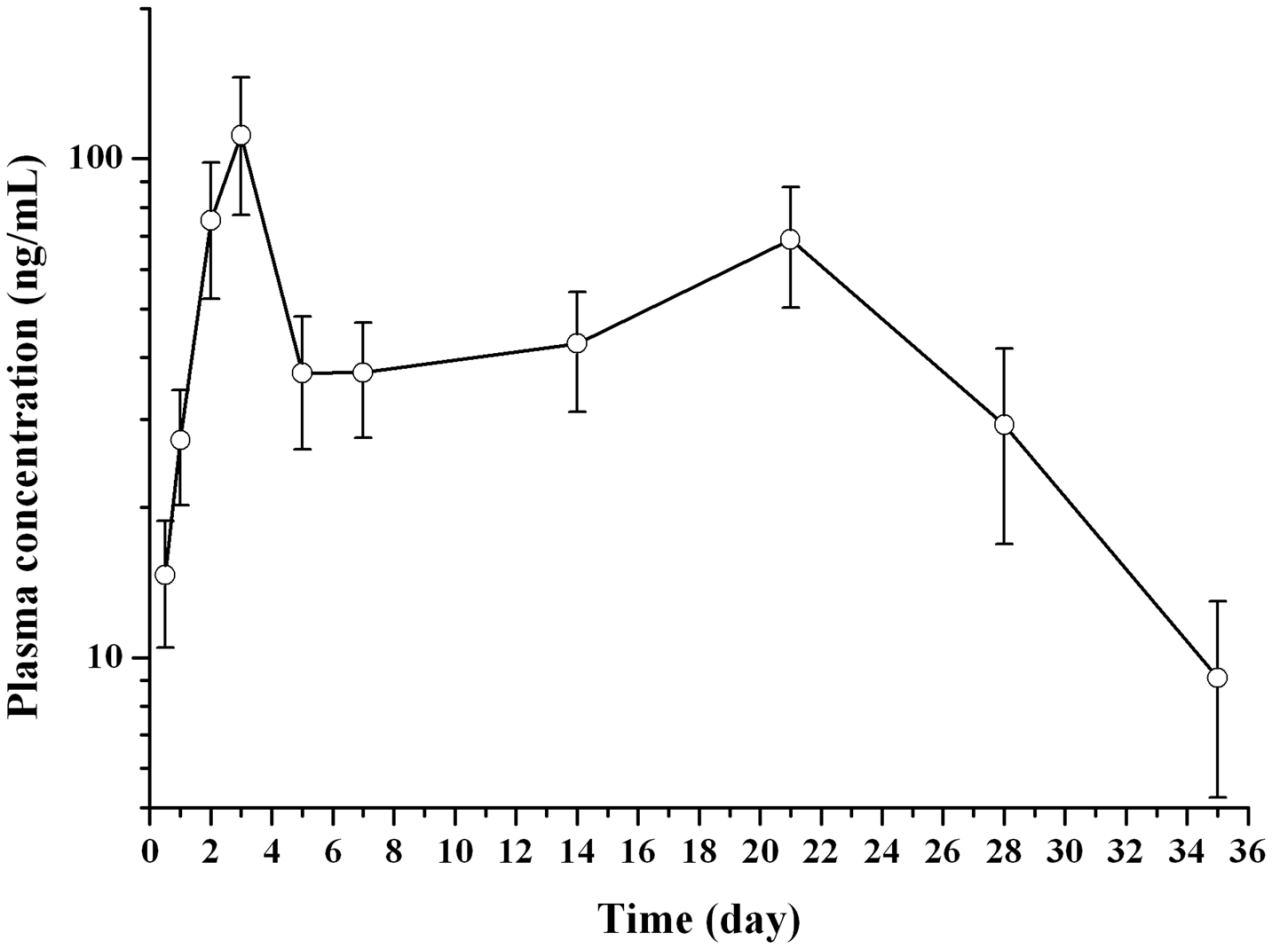
Mean plasma drug concentration-time curve of nalmefene in rats following the single subcutaneous injection of drug loaded microspheres (n = 6).

The mean plasma drug concentration versus time curves for nalmefene were shown in Figs 6 and 7, corresponding to the subcutaneous administration of nalmefene solution (3 mg/kg) and nalmefene encapsulated microspheres (90 mg/kg), respectively. As displayed in Fig 6, after the single injection of nalmefene solution, the plasma drug concentration quickly reached its maximum value (689.16 ± 181.26 ng/mL) in 2 min, and then it declined fast, left around 10% of the C_max_ value 2 h later, which implied a rapid *in vivo* elimination of nalmefene exist in rats.

In the case of subcutaneous administration of microspheres, the *in vivo* profile of which was more smooth than the nalmefene solution group (Fig 7). The plasma nalmefene concentration approached its C_max_ of 111.42 ± 34.21 ng/mL at the day 3; then, the drug concentration decreased markedly and kept stable at a value of about 35 (ng/mL) from day 5 to day 14; after that, the plasma concentration rose gradually and exhibited its second peak value (68.95 ± 18.72 ng/mL) at day 21; following that, the plasma drug concentration dropped slowly and its value below 10 (ng/mL) at the day 35. The first high plasma drug concentration of nalmefene in the earlier five days was due to the initial fast drug release from the microparticles [35]. The later high drug level may be associated with the second fast *in vivo* drug release of the microspheres, in which the microparticles break down and the degraded pieces were intake by macrophage [38,39].

The pharmacokinetic (PK) parameters of both formulations are listed in Table 1. Compared with drug solution group, the values of T_max_, AUC, t_1/2_ and MRT of microspheres group were all significantly higher. These data demonstrated that the prepared nalmefene microspheres had a sustained release feature as our previous expectation.

**Table 1:** Pharmacokinetic parameters after single subcutaneous injection of nalmefene solution and microspheres in rats (n = 6).

Parameters	Solution (3 mg/kg)	Microspheres (90 mg/kg)
T_max_ (h)	0.06 ± 0.02	76.00 ± 23.59
C_max_ (ng/mL)	689.14 ± 181.26	111.42 ± 34.21
t_1/2_ (h)	0.15 ± 0.03	3.29 ± 0.37
AUC_(0-t)_ (ng^.^h/mL)	449.75 ± 73.95	36720.45 ± 3536.16
AUC_(0-∞)_ (ng^.^h/mL)	454.68 ± 74.62	38235.32 ± 3715.72
MRT_(0-t)_ (h)	2.46 ± 0.93	366.84 ± 35.39
MRT_(0-∞)_ (h)	2.78 ± 0.96	393.12 ± 36.83

Maintaining a relative constant blood drug concetration in the long-term pharmacotherapy is the distinguished PK advantage of nalmefene loaded microspheres. Adverse effects (e.g. nausea in patients) [40] connected to peak blood levels after the bolus administration of nalmefene, might be reduced by treated with the microsphere preparation.

The peak blood drug concentration was related to the burst release *in vitro*, which is the disadvantage of microspheres preparation and needs special attention. According to the clinical study reports, the effective dose nalmefene by oral to treat insobriety was 10–80 mg/day [41,42], and nalmefene exhibits a linear pharmacokinetic profile in human [10]. Combining these two points, it might be reasonable for us to derive such a conclusion: the peak plasma concentration of 80 mg/day is approximately 8 times of that of 10 mg/day. In other words, the peak plasma concentration, equaling to 8 times of maintenance plasma concentration, is considered safe in human. As shown in Fig 7, peak plasma concentration (111 ng/mL) was about 3.2 times of maintenance plasma concentration (35 ng/mL). As blood fluctuation in rats (3.2) was smaller than 8 for microspheres, the peak plasma concentration that might produce by this depot formulation may be safe in human. However, extrapolating this point to human is just in theory and needs further studies.

The nalmefene dose delivered by the present microspheres is a bit smaller than the clinical requirement. In a clinical alcoholism intervention study, the mini effective peroral dose of nalmefene was 10 mg/day [41]. Thus, a dose of 4 mg/day may be enough for the effective drug therapy, since the bioavailability of nalmefene per oral is about 40% [14]. Considering the drug loading of the current microspheres was 6.8%, there need to inject at least 1647 mg microspheres to maintain the therapeutic blood levels for 4 weeks. The max dose of current available microspheres in the market was about 1100 mg (naltrexone microspheres) [43], so the loading ratio of present microspheres should be enhanced to reduce the injection amount. With this aim in mind, this work is still ongoing in our research team.

### *In vitro* and *in vivo* correlation

**Fig 8:**
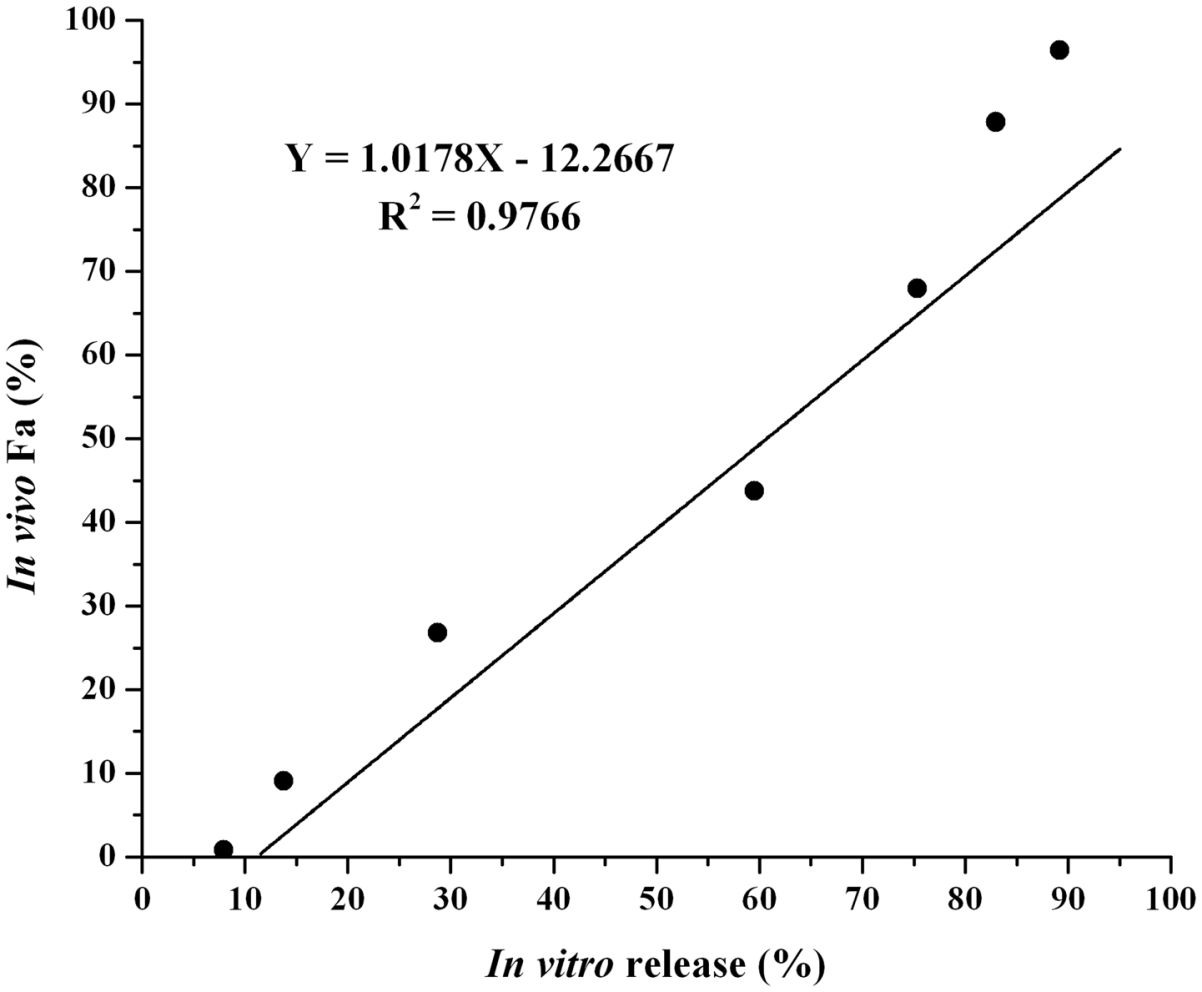
Linear regression plot for the percent released *in vitro* versus percent absorbed *in vivo*.

The data of *in vitro* drug release (%) versus *in vivo* drug absorbed (F_a_, %) for the nalmefene loaded microspheres were plotted and exhibited in Fig 8, and the linear regression equation is included. This is a point-to-point correlation study and could be classified as level A correlation according to the FDA definition [44]. Based on the value of correlation coefficient (R^2^ = 0.9766, p < 0.01), the linear regression correlation between the fraction of drug released *in vitro* and drug absorbed *in vivo* was considered good. Thus, this result indicated that the method used in the current dissolution test seemed to be rational to anticipate the *in vivo* release profiles of nalmefene microsphere and could be employed in the future quality control of such drug depot formulation [45].

### Pharmacodynamics study

Nalmefene is an opiate derivate and has the similar chemical structure and pharmacology activity as naltrexone (an opiate antagonist), thus the hot plate test used in the assessment of antinociceptive effects of naltrexone microspheres [30] could be utilized to evaluate the pharmacology effects of nalmefene loaded microspheres in this study.

In the mouse hot-plate experiment, there was no significant difference in response time (RT) for each group before the morphine challenge. Therefore, the initial status of all mice was regarded as homogenous.

**Fig 9:**
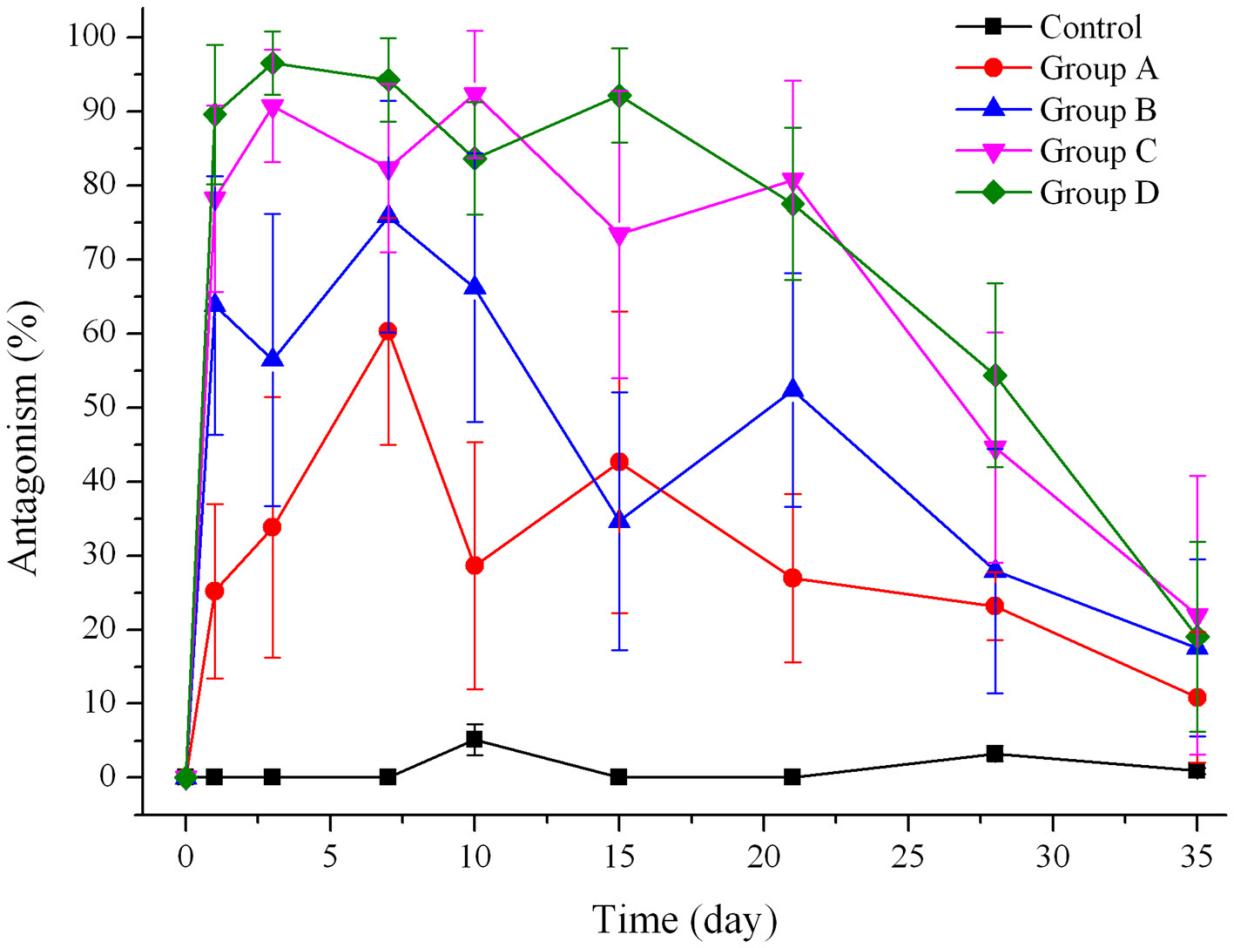
Percent analgesic antagonism-time profile of different doses of nalmefene microspheres (n = 10). Control: blank microspheres; Group A: nalmefene microspheres eq 40 mg nalmefene/kg; Group B: nalmefene microspheres eq 80 mg nalmefene/kg; Group C: nalmefene microspheres eq 160 mg nalmefene/kg; Group D: nalmefene microspheres eq 320 mg nalmefene/kg.

As shown in Fig 9, the four doses of nalmefene microspheres demonstrated antagonism to morphine analgesia for various periods. In the blank microsphere treated group, morphine (20 mg·kg^-1^) generated almost no antagonistic effect (~0%) on this hot plate test. The group A antagonized morphine analgesia with 25.19% of antagonism ratio at day 1, and reached its highest effect at day 7; then the antagonism ratio exhibited another value peak at day 15; after that the effect diminished with 10.85% at day 35. The other three groups showed a similar antagonism profile: It climbs to its maximum value from zero and then descends slowly, after a second peak value, it falls gradually. This phenomenon was speculated to be related to the *in vivo* drug release that mentioned above. However, the time of the two antagonism peaks varied as the dosage changed.

It was obviously seen in Fig 9 that the antagonism effect increased as the dosage increased. It implied that the tailored pharmacodynamics could be achieved by injecting various amounts of microspheres. The 30% antagonism ratio was usually taken as the critical point [46], antagonism value larger than that was considered as antagonism effective. The effective therapy time of Group A to D was 7, 21, 28 and 28 days, respectively. Thus, high doses of nalmefene microspheres had much more longer antagonism enduring than low doses. Furthermore, this result suggested that the prepared extended-release microspheres demonstrated a long-term antagonism effect to the morphine induced analgesia effect.

### Immunotoxicity study

A drug-induced immune response can significantly alter the safety and efficacy of drugs. Therefore, early testing and evaluation of immunogenic properties of nalmefene PLGA microspheres is essential.

Two weeks after the challenge treatment, all guinea pigs from the saline group and the two nalmefene loaded microsphere groups did not demonstrate any symptoms of allergic reaction and no one died. Thirty days later, there were also no allergic reactions observed in these guinea pigs. Whereas, all the animals in the bovine serum albumin group (positive control) showed different degrees of shivering, standing hair, sneezing, cough, wheezing, dyspnea, convulsions, incontinence and other obvious symptoms of allergic reactions, and one guinea pig died in this group.

Optical density (OD) values of dissolved skin from rats at day 1 and 30 of passive cutaneous anaphylaxis test are listed in Tables 2 and 3, respectively. OD values in the bovine serum albumin group were significantly higher (p < 0.05) than other groups, and there was no significant difference between the two nalmefene microsphere dosing groups (p > 0.05). Neither microsphere groups displayed passive cutaneous anaphylaxis symptoms at either time point, suggesting that there was no passive cutaneous anaphylaxis to nalmefene PLGA microspheres in Sprague-Dawley rats.

**Table 2:** Optical density (OD) values at day 1 after the passive cutaneous anaphylaxis test in rats (n = 4).

Group	1 : 2 dilution	1 : 8 dilution	1 : 32 dilution
Physiologic saline group (1 mL/kg)	0.0081 ± 0.0033	0.0086 ± 0.0031	0.0083 ± 0.0035
Bovine serum albumin group (10 mg/kg)	0.0224 ± 0.0087*	0.0219 ± 0.0085*	0.0216 ± 0.0091*
Nalmefene microsphere group (120 mg/kg)	0.0078 ± 0.0036	0.0072 ± 0.0032	0.0079 ± 0.0037
Nalmefene microsphere group (600 mg/kg)	0.0094 ± 0.0042	0.0089 ± 0.0038	0.0092 ± 0.0039

* p < 0.05 compared to the physiologic saline group.

**Table 3:** Optical density (OD) values at day 30 after the passive cutaneous anaphylaxis test in rats (n = 4).

Group	1 : 2 dilution	1 : 8 dilution	1 : 32 dilution
Physiologic saline group (1 mL/kg)	0.0084 ± 0.0035	0.0082 ± 0.0034	0.0081 ± 0.0029
Bovine serum albumin group (10 mg/kg)	0.0215 ± 0.0087*	0.0217 ± 0.0088*	0.0219 ± 0.0082*
Nalmefene microsphere group (120 mg/kg)	0.0079 ± 0.0032	0.0076 ± 0.0034	0.0078 ± 0.0031
Nalmefene microsphere group (600 mg/kg)	0.0098 ± 0.0044	0.0094 ± 0.0041	0.0092 ± 0.0043

* p < 0.05 compared to the physiologic saline group.

The results of above two tests demonstrated that the nalmefene microspheres exhibited no significant immunotoxicity. However, these tests reported here was not adequate, because the immunogenicity evaluation includes many other tests (humoral and cell related immune experiments). Thus further studies are in need to elucidate immunogenic properties of nalmefene microspheres, and we will publish the safety evaluation data in the following paper when the studies are completed.

## Conclusions

In this paper, the injectable PLGA microspheres containing nalmefene were fabricated by an O/O emulsion solvent evaporation method. The prepared microspheres exhibited a sustained *in vitro* release of nalmefene more than one month, which was well fitted with a first-order model. The *in vitro* drug release mechanism for this microsphere was dominated by both drug diffusion and polymer degradation. The pharmacokinetics study results from rats demonstrated that a relatively constant of nalmefene plasma concentration could be acquired for over 4 weeks, and pharmacodynamics studies confirmed that the prepared microspheres could produce a long-acting antagonism efficacy on rats. Local irritation and muscle stimulation tests in rabbits demonstrated that the nalmefene microspheres could used for subcutaneous or intramuscular administration, without causing any permanent damage to the skin or muscle. Therefore, the extended release nalmefene loaded PLGA microsphere may be a promising preparation for the long-term treatment of alcohol dependence.
